# Exploring the human archaeome: its relevance for health and disease, and its complex interplay with the human immune system

**DOI:** 10.1111/febs.17123

**Published:** 2024-03-31

**Authors:** Torben Kuehnast, Christina Kumpitsch, Rokhsareh Mohammadzadeh, Thomas Weichhart, Christine Moissl‐Eichinger, Holger Heine

**Affiliations:** ^1^ D&R Institute for Hygiene, Microbiology and Environmental Medicine Medical University of Graz Austria; ^2^ Institute of Medical Genetics Medical University of Vienna Austria; ^3^ BioTechMed Graz Austria; ^4^ Research Center Borstel – Leibniz Lung Center Division of Innate Immunity, Airway Research Center North (ARCN), German Center for Lung Research (DZL) Borstel Germany

**Keywords:** archaea, immune response, macrophage, methane, *Methanobrevibacter*, *Methanobrevibacter smithii*, methanogens, *Methanosphaera stadtmanae*

## Abstract

This Review aims to coalesce existing knowledge on the human archaeome, a less‐studied yet critical non‐bacterial component of the human microbiome, with a focus on its interaction with the immune system. Despite a largely bacteria‐centric focus in microbiome research, archaea present unique challenges and opportunities for understanding human health. We examine the archaeal distribution across different human body sites, such as the lower gastrointestinal tract (LGT), upper aerodigestive tract (UAT), urogenital tract (UGT), and skin. Variability in archaeal composition exists between sites; methanogens dominate the LGT, while Nitrososphaeria are prevalent on the skin and UAT. Archaea have yet to be classified as pathogens but show associations with conditions such as refractory sinusitis and vaginosis. In the LGT, methanogenic archaea play critical metabolic roles by converting bacterial end‐products into methane, correlating with various health conditions, including obesity and certain cancers. Finally, this work looks at the complex interactions between archaea and the human immune system at the molecular level. Recent research has illuminated the roles of specific archaeal molecules, such as RNA and glycerolipids, in stimulating immune responses via innate immune receptors like Toll‐like receptor 8 (TLR8) and ‘C‐type lectin domain family 4 member E’ (CLEC4E; also known as MINCLE). Additionally, metabolic by‐products of archaea, specifically methane, have demonstrated immunomodulatory effects through anti‐inflammatory and anti‐oxidative pathways. Despite these advancements, the mechanistic underpinnings of how archaea influence immune activity remain a fertile area for further investigation.

## Graphical abstract

This work investigates the archaeome, an essential segment of the human microbiome, focusing on its relationship with the immune system. Here, we outline the types of archaea across different body areas and underscore possible links to health issues, such as obesity and specific cancers. Additionally, we emphasize recent findings on the role of archaeal molecules and by‐products, including methane, in influencing immune reactions and their effects on immune mechanisms.

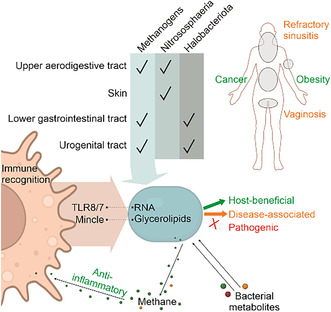

**Abbreviations**
amoA: ammonia monooxygenase subunit AmoA
AOA: ammonia‐oxidizing archaea
ASC: apoptosis‐associated speck‐like protein containing a CARD
BMDCs: bone marrow‐derived dendritic cells
BMI: body mass index
Cas9: CRISPR‐associated protein 9
CD: cluster of differentiation
CH4: methane
CRC: colorectal cancer
CRISPR: clustered regularly interspaced palindromic repeats
DC: dendritic cell
DNA: deoxyribonucleic acid
DSS: dextran sulphate sodium
FISH: fluorescence *in situ* hybridization
GTDB: genome taxonomy database
HEK293: human embryonic kidney 293 cells
HMP: Human Microbiome Project
IBD: inflammatory bowel disease
IBS: irritable bowel syndrome
IBS‐C: irritable bowel syndrome type C
IBS‐D: irritable bowel syndrome type D
IFN‐γ: interferon‐gamma
IgG: immunoglobulin G
IL‐10: interleukin 10
IL‐1β: interleukin 1 beta
IL‐6: interleukin 6
IRF3: interferon regulatory factor 3
LGT: lower gastrointestinal tract
LPS: lipopolysaccharide
MAGs: metagenome‐assembled genomes
mcrA: methyl coenzyme M reductase
MINCLE: C‐type lectin domain family 4 member E
MIP‐2: C‐X‐C motif chemokine 2
mmHg: milimeters of mercury
MPO: myeloperoxidase
MyD88: myeloid differentiation primary response protein
NF‐κB: nuclear factor NF‐kappa‐B
NLRP3: NACHT, LRR and PYD domains‐containing protein 3
NOD: nucleotide‐binding oligomerization domain proteins
NOD1: nucleotide‐binding oligomerization domain‐containing protein 1
NOD2: nucleotide‐binding oligomerization domain‐containing protein 2
PBMC: peripheral blood mononuclear cells
PCR: polymerase chain reaction
PFS: progression‐free survival
PMN: modulating polymorphonuclear
ppm: parts per million
qPCR: quantitative polymerase chain reaction
ROS: reactive oxygen species
rpoB: RNA polymerase subunit beta
rRNA: ribosomal RNA
SCFA: short‐chain fatty acids
SIBO: small intestinal bacterial overgrowth
ssRNA: single‐stranded ribonucleic acid
TACK: Thaumarchaeota, Aigarchaeota, Crenarchaeota, and Korarchaeota
TC: T cell
Th17: T helper 17 cells
TLR: Toll‐like receptor
TNF‐α: tumor necrosis factor alpha
UAT: upper aerodigestive tract
UGT: urogenital tract
UNC93B: protein unc‐93 homolog B1

## Introduction

In the ever‐evolving panorama of human biology, the human microbiome has established itself as a central focus of scientific research, particularly because of its profound impact on human health and susceptibility to disease. Beyond the spotlight on the bacterial majority, however, a discrete and relatively under‐researched ecological component, known as the human archaeome, has emerged as an interesting and maybe also central player in the human body [1, 2, 3, 4, 5, 6, 7, 8].

The human archaeome represents all archaeal microorganisms that inhabit various niches within the human body. These archaea, as a distinct domain of unicellular microorganisms, are biologically different from bacteria and eukaryotes. They were first noted for their occurrence in extreme environments such as hydrothermal vents and acidic lakes, but are now recognized as an integral part of the human ecosystem. In particular, methanogenic archaea, such as *Methanobrevibacter* species and *Methanosphaera stadtmanae*, as well as members of the Methanomassiliicoccales (Thermoplasmatota phylum), have established themselves as constant and prevalent members of the human gastrointestinal microbiome [3].

Nevertheless, the detection of archaea within the human microbiome is commonly hindered by various methodological challenges. These obstacles include difficulties in their cultivation [9], as well as molecular impediments [10]. These challenges arise primarily due to the prevailing emphasis on bacteria‐centric approaches in various aspects of the research process, such as sampling techniques, DNA extraction protocols, selection of PCR primers, and the utilization of classification databases. Consequently, the human archaeome remains often unresolved with respect to diversity and taxonomic classification, and particularly their function and mechanistic role in human health and disease.

Despite their differential abundance in disease patterns, mechanistic roles of archaea and their products in the human body have only rarely been investigated, and their interplay with the human immune system has opened novel questions on method of action [11].

In this review, we will summarize the current knowledge on the human archaeome from various body sites and their potential role in disease (see also [3, 4, 8, 11, 12, 13, 14, 15, 16]), but will specifically focus on the archaea‐immune system interplay and the current status of knowledge.

## Archaea at different body sites in health and disease

The discrete niches of the human body can be divided into several unique body sites, namely the upper aerodigestive tract (UAT), the lower gastrointestinal tract (LGT), the urogenital tract (UGT) and the skin. With conditions strongly varying, different types of archaea thrive in each system.

Aerated areas, such as the skin and the nostrils area of the nose, are thinly populated by ammonia‐oxidizing archaea (AOA) of the class Nitrososphaeria (formerly: Thaumarchaeota), with a skin O_2_ concentration of approximately 145 mmHg on the surface [17]. It is assumed that these AOA use the ammonia compounds of sweat and skin (e.g. urea) to carry out nitrification, i.e. to oxidize ammonia under aerobic conditions and produce nitrite in the process [18]. Nitrification is accompanied by a lowering of the pH value and could, therefore, have a positive effect on the skin barrier (general skin pH: 5.3–6.5; [17]). The archaea on the skin are considered beneficial at this stage of the research, but further analysis is required. It is noteworthy that the abundance of archaea is particularly increased in children and the elderly, which may be attributed to physical and chemical changes of skin physiology [19].

Methanogens, i.e., methane‐producing archaea, are usually found in anoxic niches. Using bacterial metabolites such as acetate, formate, H_2_, CO_2_, or methyl compounds, they ‘complete’ fermentation by converting them into CH_4_, which leaves the body through flatus or exhalation. The overwhelming number of methanogens are found in the large intestine (pH 6.7, 37 °C, O_2_ concentration: 0.5–11 mmHg; [17]), where they interact syntrophically with numerous bacteria and make their metabolism more efficient by consuming their end‐products. The methane produced has a direct effect on the human body, as it slows down intestinal motility, which is often associated with constipation (see below). Methanogens also thrive in anoxic niches in the oral/nasal cavity (e.g., in connection with sinusitis or periodontitis) or in the urinary tract [8]. There, they can also be involved in pathogenic actions, which will be discussed later.

With the LGT containing 99% of all microbial biomass in humans [20] and at the same time being the gateway for a multitude of diseases, research on human‐associated archaea (as well as bacteria) has primarily focused on the analysis of stool samples. Consequently, our understanding of these microorganisms in other body sites, such as the airways and lungs, remains limited. Although archaeal signatures, including Woesearchaeales spp., have been found in these areas of the body, their taxonomy, physiology, and function remain largely unknown to date [17].

**Fig. 1:**
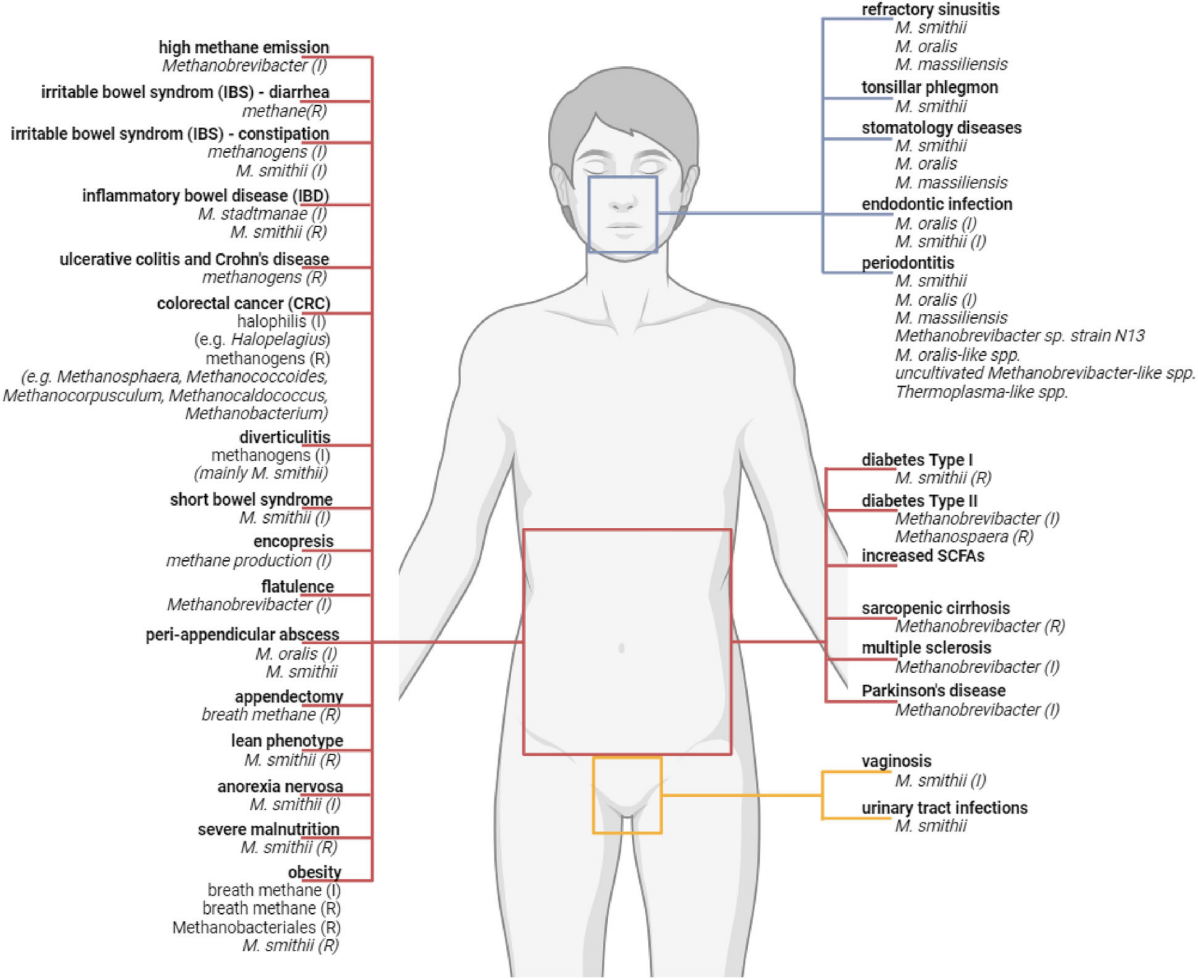
Diseases associated with altered archaeal composition. Blue – oral samples; red – stool samples, yellow – urogenital samples; I, increased; R, reduced. Illustration was created with BioRender.com.

Although archaea, so far, have never met terms of a pathogen, literature does indeed describe a variety of correlations and associations of archaea with health and disease (Summarized in Fig. 1, Table 1; for more details, see also [8]).

**Table 1:** Association of methanogens and different human host phenotypes. Only studies published after 2022 are included. For earlier reports refer to [8].

Host phenotype	Study type	Study size	Detection of	Method	Relevance	References
Irritable bowel syndrome (IBS)	Randomized controlled trials	*n* = 47 IBS‐D (diarrhea‐predominant) patients, *n* = 124 IBS‐C (constipation‐predominant) patients	*M. smithii*	Breath methane, 16S rRNA gene sequencing	Compared to IBS‐D patients, IBS‐C subjects were characterized by higher abundance of methanogens, especially *M. smithii* as well as high breath methane	[21]
	Longitudinal	*n* = 27 controls, *n* = 55 IBS patients	*Methanobrevibacter*	qPCR, 16S rRNA gene sequencing	No difference between the abundance of *Methanobrevibacter* between IBS patients and healthy adults. Significantly higher alpha diversity in microbiome of subjects with increased methanogen load compared to healthy subjects	[22]
Urogenital infection	Prospective	n = 373 subjects without urinary tract infection	Methanobacteriota, Thermoproteota, Halobacteriota	16S rRNA gene sequencing, FISH	This study suggested archaea as the usual colonizers of the human urinary tract and not as an indicator of urinary infection.	[23]
Small intestinal bacterial overgrowth (SIBO)	Restrospective, cross‐sectional	*n* = 977 control, *n* = 484 SIBO positive patients	Methanogens	Breath test	Patients with methanogen SIBO showed different symptoms compared to those caused by hydrogen‐producing bacteria and in particular the symptoms are with lower incidence of vitamin b12 deficiency	[24]
Metabolic disorders	Prospective, randomized, open, controlled trial	*n* = 162 (83 pet owners, 79 non‐pet owners) patients with conventional treatment for coronary heart disease	*Methanobrevibacter*	16S rRNA gene sequencing	The presence of *Methanobrevibacter* in the samples of dog owners might explain the lower prevalence of metabolic syndrome and obesity in this population	[25]
Tumor	Prospective	*n* = 3591 saliva samples, *n* = 128 stool samples, 1859 HMP website	*Euryarchaeota* and the TACK superphylum, in particular methanogenic archaea, such as *Methanobacteria*	Shotgun metagenome sequencing	The presence of genes encoding for cancer‐related metabolites in archaea using protein annotation of the archaeal MAGs	[26]
Colorectal cancer	Case–control	*N* = 17 CRC patients and *n* = 13 healthy controls	*Methanobrevibacter*, *Methanomassilicoccus*	16S rRNA gene sequencing	High abundance of *Methanobrevibacter* *Methanomassilicoccus* in the colon of CRC patients compared to healthy controls suggesting that methanogens may be involved in CRC development	[27]
Steatohepatitis	Case–control	*n* = 10 controls, *n* = 10 subjects with non‐alcoholic steatohepatitis	*M. smithii*	qPCR, 16S rRNA gene sequencing	A decreased frequency of *M. smithii* was observed in subjects with non‐alcoholic steatohepatitis. This study suggested that following the depletion of *M. smithii*, dysbiosis can occur	[28]
Celiac disease	Case–control	*n* = 12 celiac disease patients, *n* = 8 subjects with a gluten‐free diet, *n* = 9 controls	*Methanobrevibacter*	16S rRNA gene sequencing	Decreased abundance of *Methanobrevibacter* was found in subjects with celiac disease. This study suggested the potential progression of celiac disease following the decreased abundance of *Methanobrevibacter*	[29]

### Upper aerodigestive tract (UAT)

The upper aerodigestive tract (UAT) comprises the upper respiratory and digestive systems, including the nose, mouth, and pharynx, providing aerobic and anaerobic niches lined with mucus [30]. Various microorganisms, including archaeal taxa, especially *Methanobrevibacter*, colonize this diverse and moist habitat. While archaea are present in both healthy and diseased individuals, they may promote the growth of specific microbial networks rather than directly causing diseases in certain conditions of the UAT [31].

Refractory sinusitis, which involves persistent inflammation of the sinuses, is characterized by the presence of anaerobic microbes colonizing the affected area [32]. A case series study by Sogodogo *et al*. [32] identified and cultured various archaea strains, including *Methanobrevibacter* species, in pus samples obtained from patients with refractory sinusitis. Among the archaeal strains detected were *Methanobrevibacter smithii*, *Methanobrevibacter oralis*, and *Methanobrevibacter massiliense*. Moreover, pus samples from another UAT disease, namely tonsillar phlegmon (case report) revealed *M. smithii* signatures via 16S rRNA gene sequencing approach [33].

In oral health conditions (stomatology diseases), *Methanobrevibacter* species are detected regardless of the specific oral region. In periodontitis patients, *M. massiliense*, *M. oralis*, *M. smithii*, and other related species (uncultivated *Methanobrevibacter*‐like species, as well as *Thermoplasma*‐like species) are found in subgingival plaques [34, 35, 36] and deep periodontal pockets [36]. These methanogens coexist with anaerobic and sulfate‐reducing bacteria (e.g. *Prevotella intermedia* [37] or *Desulfovibrio* [38]), indicating a symbiotic relationship where methanogens utilize bacterial by‐products to support their own growth and that of the bacteria [34]. This interaction may contribute to the development and severity of periodontitis [39], especially *M. oralis*, which is more prevalent in deep pockets of patients compared to healthy individuals [40, 41].

In addition, increased abundances of *M. oralis* and *M. smithii* such as in inflamed pulp tissue in endodontic infections were also detected by amplicon sequencing in other oral cavity infections [42].

### Urogenital tract (UGT)

The urinary and genital tract (UGT), which are closely interconnected, possess shared anatomical features. Typically, these regions exhibit a moist and warm environment with a certain level of acidity (urinary tract: pH 4.5–8.6, O_2_ concentration: 0.47–51.5 mmHg, Temp: 36.9–39.9 °C; vagina: pH 4.2–5.0, O_2_ concentration: 15–35 mmHg, Temp: 37 °C; [17]). They serve as a habitat for specific microbial species, albeit with lower diversity, primarily dominated by lactobacilli. Due to the close proximity between the urinary and gastrointestinal tracts, it is plausible that microorganisms can be transmitted from the latter via feces, subsequently leading to urinary tract infections and vaginosis. Consequently, it is conceivable that even the most prevalent archaeon found in the gut, namely *M. smithii*, could potentially colonize the UGT. In fact, a limited number of studies have observed an increased presence of *M. smithii* in vaginal samples obtained from patients with vaginosis through microscopic examination, isolation, and sequencing techniques [33, 43]. Furthermore, a prospective study analyzing 383 urine samples identified *M. smithii* as part of the urinary tract microbial community. Together with other enteric bacteria, its presence may contribute to the development of urinary tract infections [43].

### Skin

Archaeal signals frequently remain either at or below the threshold of detection (e.g. observed in [44]), thereby impeding a comprehensive understanding of the potential role played by archaea within the human skin microbiome.

However, despite their relatively low prevalence, archaeal signals were identified in all 13 human subjects examined through 16S rRNA gene sequencing, as well as through the detection of the archaeal *amoA* gene, which encodes the essential ammonia monooxygenase enzyme involved in ammonia oxidation [18]. Notably, these archaeal signals were linked to Nitrososphaerales taxa (formerly classified as the Thaumarchaeota phylum, now categorized as Thermoproteota in the GTDB taxonomy), and these signatures were consistently present in all human subjects. Moreover, these Nitrososphaerales archaea demonstrated a close phylogenetic affinity to those previously identified in clean room environments and intensive care units [45]. Although the precise physiological relevance of these ammonia‐oxidizing archaea (AOA) to skin remains a subject of conjecture, their presence has been associated with human age and skin physiology [19]. Despite the identification of archaeal signals on the skin, which have recently been reclassified as belonging to the *Nitrosocosmicus* genus and other unknown genera within the Nitrososphaeraceae family, through a range of methodologies including archaeal 16S rRNA gene and *amoA* cloning, fluorescence *in situ* hybridization [18], next‐generation sequencing, and infrared hyperspectral imaging [19], a multitude of unresolved questions persist. These inquiries pertain to various aspects, including (a) the abundance of these archaeal populations, (b) their longitudinal stability over time, (c) their functional roles and metabolic activities, (d) their integration within the broader bacterial microbiome, and (e) their potential associations with human health and physiological processes.

### Lower gastrointestinal tract (LGT)

The lower gastrointestinal tract (LGT), from the small intestine to the rectum, serves as the major habitat of human‐associated archaea. Depending on the methods of DNA isolation (bead beating, lysosome addition, duration), targeted genetic regions (whole genome, 16S rRNA, *rpoB* or *mcrA* genes) and way of amplification (primer choice, nested PCR), results of archaeal detection can vary strongly.

Kim *et al*. [46] detected archaea in 42.7% of 897 East Asian subjects based on 16S rRNA gene amplicon sequencing. Once detected, the relative abundance in the gut ranged from 2% to up to a maximum of 15% [46]. Mohammadzadeh *et al*. [8] referred to a metagenomic approach and identified *M. smithii* in 91.32% of 691 datasets from various geographic locations (Europe, Asia, North/South America, Africa, and Oceania). The relative abundance of *M. smithii* was described to be 0.56% for all individuals [8]. Others used a combined 16S rRNA and *rpoB*‐based method and detected *M. smithii* in 95.7% and *M. stadtmanae* in 29.4% of 700 individuals [47].

The main archaeal representatives in the LGT thus are the Methanobacteriales, which comprise the most abundant methanogenic species *M. smithii*, as well as the newly discovered *Candidatus M. intestini* [48]. These two species constitute up to 94% of the human gut archaeome [49]. Other relevant archaeal members of the gut include *M. stadtmanae*, *M. oralis*, Methanosarcinales [49], Methanomassiliicoccales [50], and Haloarchaea [46, 51].

Although the exact role of archaea in the complex network of gut microbiome is still to be fully elucidated, the methane‐producing representatives occupy a niche at the very end of the microbial gut metabolism. By being the only group to perform methanogenesis, methanogenic archaea convert bacterial end‐products, such as hydrogen and carbon dioxide, acetate and methyl compounds, into methane and energy. Methane moves through the gut lumen and leaves the body via flatus (75%, 7 mmol per day), or enters the blood through the intestinal wall, finally reaching the lung (25%, 2.3 mmol per day), where it can be detected in the breath [52]. High methane breath emission (> 5 ppm) has been linked to an approx. 1000‐fold increase of *Methanobrevibacter* abundance in the gut [53]. With this in mind, methane production represents a reliable biomarker for the presence of methanogens in the gut.

Higher or lower breath methane levels (and the corresponding variation in abundances of methanogens) have been associated with various health conditions. Below, we exemplarily list medical conditions, for which associations of methanogens have been described. For a full overview of observations please see Table 1 (an updated table from Mohammadzadeh *et al*. [8]), and the key findings summarized in Fig. 1.

In the LGT, Djemai *et al*. [54] found two *Methanobrevibacter* strains (*M. oralis* and *M. smithii*) to be increased in the appendix of four people suffering from peri‐appendicular abscesses. Their role and involvement in the pathology of the abscesses remains unknown. Interestingly, appendectomy was able to reduce the methane levels measured in breath indicating that from a general perspective of gut‐diversity, the appendix might be a reservoir for methanogens [55]. Unfortunately, a recent metagenome‐based study focusing on appendicitis could not reveal meaningful signatures of methanogenic archaea, and faced technical issues [56].

Methanogens have also been connected to slow intestinal transit time and constipation, as it could be causally attributed to higher methane production [57]. This effect was also shown in irritable bowel syndrome (IBS)‐patients, where higher prevalence of methanogens (especially *M. smithii*) was significantly associated with constipation (type C patients). Lower prevalence of *M. smithii* was correlated with type D patients who suffered from diarrhea instead [58]. A recent study by Onana Ndong *et al*. [59] further investigated connections of small intestinal bacterial overgrowth (SIBO), methane and hydrogen production and showed that methane levels were significantly higher in constipated patients, whereas in diarrheal IBS, hydrogen levels were instead significantly elevated. IBS‐severity was unaffected by either hydrogen or methane production. Furthermore, Leiby *et al*. [60] summarized own and other studies showing that children suffering from encopresis (fecal incontinence) and constipation showed higher prevalence of SIBO and were more likely to be methane‐producers than children with other gastrointestinal disorders. Furthermore, higher methane levels correlated significantly with higher radiographic fecal impaction scores [60].

Scanlan *et al*. [49] showed that in their analyses no significant differences of methanogens could be identified. In their study design, they did not differentiate between IBS‐D and IBS‐C, which illuminates the importance of metadata to be incorporated into datasets. Their analyses, however, did see the abundance of *M. smithii* dropping when diagnosed with inflammatory bowel disease (IBD) from the range of 45–50% to 24% (Ulcerative colitis, *P* < 0.01) or 30% (Crohn's disease, *P* < 0.1) [49]. These results are supported by Ghavami *et al*. [61], who found out that abundances of *M. smithii* were significantly lower in IBD‐patients compared to healthy controls. These studies seem to indicate that an inflammatory state may correlate with less methanogens.

Million *et al*. [62] quantified the abundance of *M. smithii* in obese people and showed that it was significantly reduced compared to non‐obese controls. Goodrich *et al*. [63] summarized their and other work to show that *M. smithii* was associated with a lean phenotype. At the same time, higher breath methane breath levels in combination with higher hydrogen production showed the opposite result and correlated significantly with an increased BMI. The BMI of the group showing only increased methane production was significantly lower and comparable to other groups, such as those without any increased production or only increased hydrogen production [64]. If it is not only about methane, but methane and hydrogen to describe and explain phenotypes correctly, this may point at the importance of microbial networks of methanogens to be investigated in more detail in the future.

Coker *et al*. [65] differentiated between moderate and advanced colorectal cancer (CRC) and showed that patients with advanced CRC had an enrichment of halophilic archaea (*Halopelagius*) and a reduction of methanogens, i.e., *Methanosphaera*, *Methanococcoides*, *Methanocorpusculum*, *Methanocaldococcus* and *Methanobacterium* species. Liu *et al*. [66] looked at patients with non‐small cell lung cancer, which is not directly connected to the gut, and compared the microbiomes of those with long or short progression‐free survival (PFS). They identified *M. smithii*, as well as one of its core enzymes of the methanogenesis—methyl coenzyme M reductase—to be enriched in patients with long PFS. Nomura *et al*. [67] conducted a similar study, showing that high concentrations of short‐chain fatty acids (SCFAs) such as propionic acid, butyric acid and valeric acid, were significantly associated with long PFS. Connecting both, it may be tempting to hypothesize that methanogens and their associated networks and metabolites change the microbial and metabolic formula of the host towards a more beneficial one.

## Archaea and the immune system

As described in previous chapters, the human archaeome has important roles in the modulation of health and disease of the human host. In order to determine the underlying functional factors for these modulations, it is pivotal to delve into the interaction of archaea and the immune system, in particular at molecular levels.

The structural and molecular traits of archaea, which differentiate them from bacteria, include the absence or alteration of certain (highly immunogenic) bacterial surface structures, like lipopolysaccharide (LPS), lipoproteins, and peptidoglycan, among others. For all the abovementioned bacterial membrane components, receptors on human immune cells have eventually been identified, such as Toll‐like receptors 4 (TLR4) for LPS [68], TLR2 for lipoproteins [69, 70] and nucleotide‐binding oligomerization domain proteins (NOD), NOD1 and NOD2, for peptidoglycans from Gram‐positive and ‐negative bacteria [71]. Until recently, information on if and how archaeal surface structures or other integral components induce signaling pathways in the human immune system has mostly been elusive.

An early report on the detection of archaea by the human immune system was by Yamabe *et al*. [72], where they were able to detect IgG antibodies against *M. oralis* in sera from periodontitis patients. Later on, this reactivity could be more specified to group II chaperonins from *M. oralis* [73]. The first immune reaction towards whole cell preparations of archaea was shown by Blais‐Lecours *et al*. [74]. In order to study pulmonary immune responses, preparations of *M. smithii* and *M. stadtmanae* were intranasally instilled in a mouse model. Although both archaeal species induced a similar IgG response, *M. stadtmanae* showed a substantially stronger immunogenicity in terms of histopathological alterations as well as accumulation of leukocytes. These studies were later extended [75] and it was demonstrated that the lung mucosal response was predominantly a Th17 inflammation reminiscent of a type IV hypersensitivity response. For *M. stadtmanae*, this response was independent of eosinophils or mast cells.

The production of antimicrobial peptides is a fundamental part of the immune system's defense response towards microbes [76]. Given the structural differences of the bacterial and archaeal cell membrane, it was unclear if archaea were susceptible to the activity of antimicrobial peptides as well. Bang *et al*. [77, 78] compared different methanogens with respect to their sensitivity towards derivatives of human Cathelicidin antimicrobial peptide, LL32 and LL20, and antimicrobial peptide NK‐lysin. The tested methanogens, *M. smithii*, *M. luminyensis*, and *M. stadtmanae*, displayed differential sensitivity with *M. smithii* being the most sensitive one, clearly indicating that the release of antimicrobial peptides by human innate immune cells is not only directed against bacteria and fungi but archaea as well.

So far, the abovementioned studies have shown humoral, antibody‐dependent detection of archaea by the mouse and human immune system. The question of whether any of the archaeal surface or internal structures are directly recognized by the innate immune system was not addressed until a decade ago. Here, two studies clearly showed that *M. smithii* and *M. stadtmanae* are detected by human peripheral blood mononuclear cells (PBMCs) [79] and dendritic cells [80]. In both studies, a marked difference in terms of the potential to stimulate immune cells could be seen: the *M. stadtmanae*‐induced cytokine induction (e.g., tumor necrosis factor alpha [TNF‐α] or interleukin‐1 beta [IL‐1β]) and the upregulation of the costimulatory molecules CD86 and CD197 was drastically higher than that of *M. smithii*; a difference in activity that had already been noticed in the studies mentioned above. Bang *et al*. [80] further demonstrated that the activation of dendritic cells was crucially dependent on phagocytosis of archaeal cells and acidification of phagolysosomes (which is a prerequisite for processing the phagolysosomal content). Thus, these results strongly hinted towards not only an intracellular recognition process, but also that the recognized archaeal structure presumably is an internal one. In additional experiments, the authors sought to identify the nature of that structure as well as its human receptor. However, using transient transfections of common TLR and NOD receptors in a human epithelial cell line (human embryonic kidney 293 cells [HEK293]), all attempts failed at that time.

**Fig. 2:**
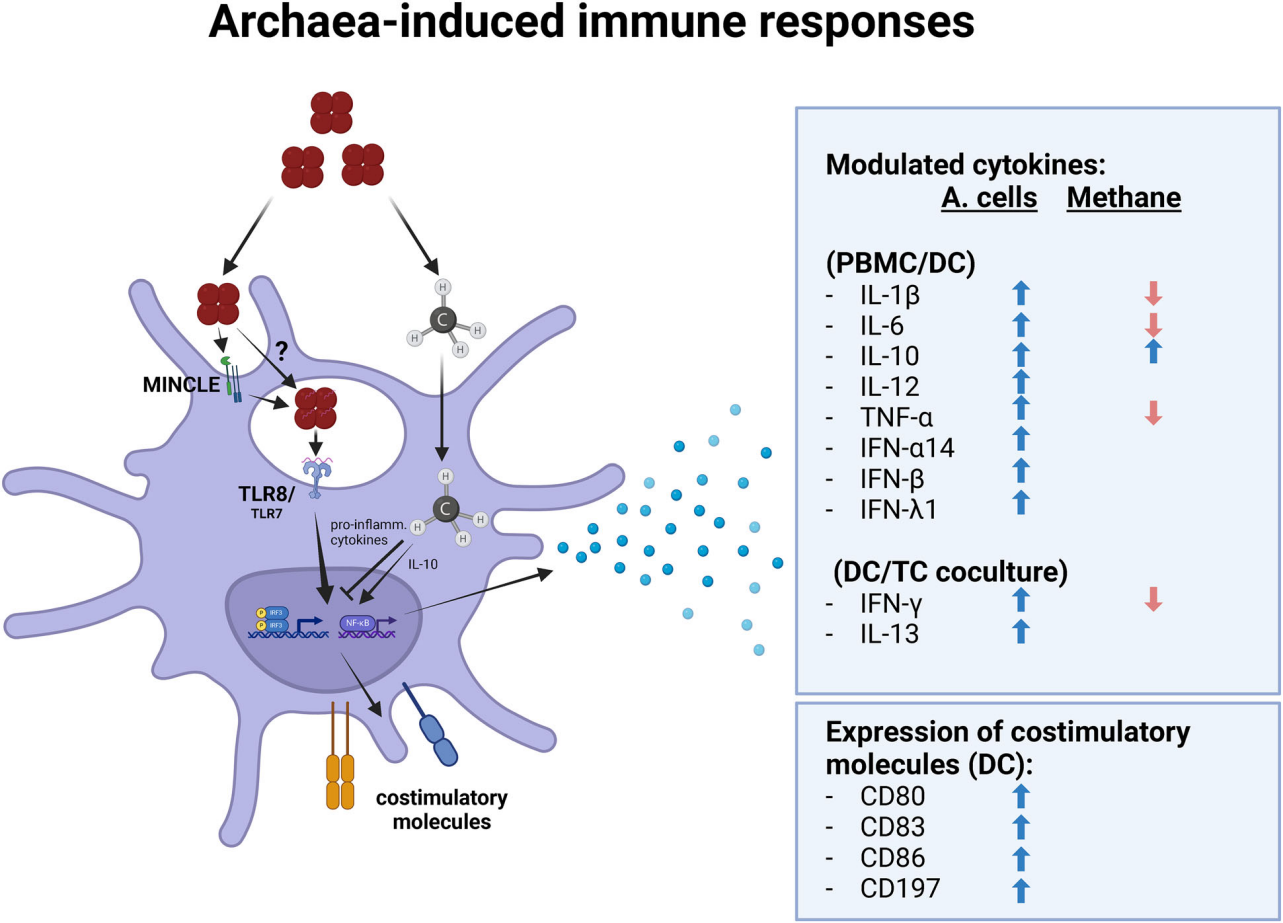
Archaea‐induced immune responses in mammalian cells. Archaea may be detected by MINCLE and TLR8, activating transcription factors such as IRF3 and NF‐κB, expressing costimulatory molecules and cytokines (see boxes). Methane may reduce proinflammatory cytokines and increase IL‐10 expression. A. cells, Archaea cells; CD, cluster of differentiation; DC, dendritic cell; IFN, interferon; IL, interleukin; IRF3, interferon regulatory factor 3; MINCLE, C‐type lectin domain family 4 member E; NF‐κB, Nuclear factor NF‐kappa‐B; PBMC, peripheral blood mononuclear cell; TC, T cell; TLR8, Toll‐like receptor 8; TNF, tumor necrosis factor. The indicated receptors, pathways, and molecules are summarized and based on published information in the referenced papers in this work and thus, may not be engaged by all archaeal species. Illustration was created with BioRender.com.

Finally, Vierbuchen *et al*. [81] discovered the first archaeal molecule recognized by a human innate immune receptor. After realizing that preparations of *M. stadtmanae* not only induce a substantial release of proinflammatory cytokines but also a strong antiviral type I and type III interferon response, they identified archaeal RNA as inducer and Toll‐like receptor 8 (TLR8) as innate immune receptor essential for this response (Fig. 2). Furthermore, using a CRISPR/Cas9‐based approach in a human monocytic cell line, they were able to identify many components (such as ‘myeloid differentiation primary response protein’ [MyD88], ‘protein unc‐93 homolog B1’ [UNC93B], ‘NLRP3, NACHT, LRR and PYD domains‐containing protein 3’ [NLRP3], and ‘Apoptosis‐associated speck‐like protein containing a CARD’ [ASC]) of the archaea‐induced signaling pathway, in particular for the archaeal RNA‐dependent activation of an alternative inflammasome pathway. This peculiar RNA‐dependent recognition through TLR8 is reminiscent of the molecular mechanism by which the allergy‐protective Gram‐positive cowshed strain *Lactococcus lactis* G121 is detected [82]. Intriguingly, also the occurrence of *M. stadtmanae* has been shown to be inversely correlated with childhood asthma [83].

Is the RNA‐dependent recognition of archaea restricted to methanogens or does this mechanism apply to all archaea? Although they did not specifically address this question, Krawczyk *et al*. [84] demonstrated that halophilic archaea can also activate human dendritic cells showing a very similar response pattern as methanogens. They could show the upregulation of costimulatory molecules such as CD80, 83, and 86 as well as the release of proinflammatory cytokines (Fig. 2). Since the preparation of the halophilic archaea did not involve procedures that would disrupt RNA integrity, activation of dendritic cells by these archaea could actually employ the same mechanisms as methanogens.

Recently, another archaeal molecule has been identified to serve as an activating structure in murine cells: archaeal glycerolipids, which are structurally different from bacterial glycerolipids by way of their ether‐linked lipids, were shown to be detected by ‘C‐type lectin domain family 4 member E’ (CLEC4E, also referred to as MINCLE), a C‐type lectin receptor present on various innate immune cells (Fig. 2). Oka *et al*. [85] used various glycerolipids based on methanogenic archaeal glycerolipids of *M. smithii* and *M. stadtmanae* and elegantly showed that the presence of a polar headgroup and a branched chain structure is essential for their activity. Furthermore, the innate immune response to the archaeal glycerolipid was largely dependent on the expression of MINCLE. Interestingly, the only natural compound used in their study, archaeol, did not show any activity.

Do these data contradict the previous finding that RNA is the essential archaeal structure activating innate immune cells? Not necessarily, due to a number of differences in their experimental systems. Oka *et al*. [85] did not use whole archaeal preparations to investigate MINCLE‐dependent immune responses. In the publication of Vierbuchen *et al*. [81], murine bone marrow‐derived dendritic cells (BMDCs) lacking Toll‐like receptor 7 (TLR7, the only functional murine receptor of ssRNA) were unable to produce any interleukin 6 (IL‐6) upon stimulation with whole *M. stadtmanae*. Surprisingly, while archaeal glycerolipids could induce the release of TNF‐α or ‘C‐X‐C motif chemokine 2’ (MIP‐2) in a MINCLE‐dependent manner, they failed to induce the release of IL‐6 in either wildtype or MINCLE‐knockout cells. Thus, MINCLE could be involved in a particular set of responses to archaea, especially in initiating the uptake of archaea in innate immune cells.

Aside from their surface structures, archaea influence the activity of the immune system through their metabolites, in particular through methane. Summarizing the efficacy of methane from a review of Jia *et al*. [86], the immunomodulatory effects seem to be based mostly on anti‐oxidative and anti‐inflammatory mechanisms. In particular, methane counteracted murine liver injury via increased PI3K/Akt/GSK‐3β‐mediated interleukin‐10 (IL‐10) expression [87]. In another study, methane could reduce the levels of TNF‐α, interferon‐gamma (IFN‐γ), IL‐6 and interleukin 1β (IL‐1β), while increasing, again IL‐10 in mice challenged with autoimmune hepatitis [88]. Boros *et al*. [89] demonstrated anti‐inflammatory effects of methane by significantly mitigating ischemia/reperfusion‐induced oxidative and nitrosative stress, reducing tissue reactive oxygen species (ROS), myeloperoxidase (MPO) activity, and modulating polymorphonuclear (PMN) leukocyte activation. Furthermore, methane showed to be reducing the inflammatory reactions in mice by decreasing the concentration of proinflammatory cytokines such as TNF‐α and IL‐6, and increasing anti‐inflammatory IL‐10 in both *in vivo* and murine macrophages in cell culture. These findings extended to a decrease in dextran sulphate sodium (DSS)‐induced colitis [90]. Taken together, methane as a terminal metabolic product of methanogens in the human gut induces increased production of IL‐10 and decreased production of IL‐6, TNA‐α, IFN‐γ, IL‐6, and IL‐1β (Fig. 2).

In conclusion, the last years provided us with a number of highly interesting findings regarding the molecular mechanisms of recognition of archaea and the subsequent innate immune responses. However, to get a complete picture of the importance and significance of immune system activation by archaea, a great deal of research is still needed.

## Conclusion

The human body and its unique body sites are inhabited by multiple, highly complex microbiome systems. In this study, we aimed to demonstrate that, in addition to bacteria, several archaea have been identified and studied, occupying important ecological niches and exerting significant influence on human health. In the UAT in conditions like refractory sinusitis and oral diseases, archaeal species such as *Methanobrevibacter* are observed, often in symbiotic relationships with anaerobic bacteria, indicating potential roles in disease etiology and progression. In the UGT, *M. smithii* has been identified in both vaginal and urine samples, indicating its possible role in conditions like vaginosis and urinary tract infections. The skin microbiome presents a more enigmatic case; although archaeal signals are generally low, they are universally present across subjects. Specifically, *Nitrososphaerales* taxa have been detected, and their physiological relevance to skin health and age remains under investigation. In the LGT, archaeal interaction with the human host seems to be based on a reduced immunogenicity. The only naive receptor to detect methanogens has just recently been identified (MINCLE). The reaction towards archaea differed from classic bacterial responses through receptors for Gram‐positive and Gram‐negative bacteria (like TLRs). Once inside phagolysosomes, only the archaeal RNA of *M. stadtmanae* induced a proinflammatory response, while *M. smithii* remained more inert. Simultaneously, methane, one of the final products of energy metabolism in an anaerobic environment, spreads systematically throughout the human body and soothes the immune system by dampening the release of IL‐6 and TNF‐α, and increasing release of IL‐10. Seemingly, the immune system did not evolve to carry heavy defense mechanisms against archaea. Although archaea were seen in connection with diseases, no pathogenic causal relations were shown so far. One may postulate that archaea are tolerated in the gut based on a purpose, which could be generating and maintaining a microbial network evolved to be beneficial for the human host. Finding these connections may be a crucial task for future research.

## Conflict of interest

The authors declare no conflict of interest.

## Author contributions

TK, CK, RM, CM‐E, and HH wrote the manuscript. TW gave essential conceptual advice and input. All authors edited the manuscript and approved the submitted version.
